# The FLOSS Project: study protocol for a parallel-group, two-arm randomized controlled trial examining the efficacy of telehealth in promoting oral and systemic health in cancer survivors

**DOI:** 10.1186/s13063-025-09247-1

**Published:** 2025-11-24

**Authors:** Chiranjeev Dash, Danyel I. Smith, Naaria Williams, Chris Leby, Newton Nyirenda, Kepher H. Makambi, Lucile Adams-Campbell

**Affiliations:** 1 Georgetown Lombardi Comprehensive Cancer Center, 1010 New Jersey Ave SE, Washington DC, 20003 USA; 2 Department of Epidemiology, Graduate School of Arts and Sciences, Georgetown University, 3520 Prospect St NW, Ste 400, Washington, DC 20057 USA

**Keywords:** Oral health, Systemic health, Cancer survivors, Telehealth, Clinical trial

## Abstract

**Background:**

The oral health of cancer survivors can be compromised by cancer treatment, resulting in reduced treatment efficacy and poorer quality of life. Data suggests that targeting oral health may improve systemic health outcomes in cancer survivors. The Facilitated Lessons on Oral and Systemic Health in Survivors (FLOSS) Project is a 2-arm, 6-month randomized controlled trial (RCT), purposed to answer the research question, “*What is the role of oral telehealth in yielding more favorable oral and systemic health outcomes in cancer survivors*?”.

**Methods:**

Participants (*N* = 100) will be recruited and randomized to the telehealth intervention (THI; *n* = 50) or usual care (UC, *n* = 50) arm. THI participants will engage in six monthly sessions designed to increase oral health-related knowledge and self-efficacy. Sessions utilize motivational interviewing and rely on behavior change techniques including: self-monitoring, goal-setting and implementation planning. Assessments of oral and systemic health outcomes will be conducted at baseline, 6- and 12-months and consist of: intraoral images, blood and saliva collection, and questionnaires to assess quality of life, lifestyle behaviors, and demographics.

**Discussion:**

FLOSS Project is one of the first theory-driven, randomized controlled trials examining the role of oral telehealth in promoting oral and systemic health in cancer survivors and is responsive to national health and research priorities. Successful completion of this project will provide evidence for an accessible intervention to promote oral and systemic health from an integrative lens in cancer survivors, post primary treatment.

**Trial registration:**

ClinicalTrials.gov; NCT06315855. Registered on 03/11/2024.

## Administrative information

Note: the numbers in curly brackets in this protocol refer to SPIRIT checklist item numbers. The order of the items has been modified to group similar items (see http://www.equator-network.org/reporting-guidelines/spirit-2013-statement-defining-standard-protocol-items-for-clinical-trials/).

**Table:** 

Title {1}	A parallel-group, two-arm randomized controlled trial examining the efficacy in telehealth in promoting oral and systemic health in cancer survivors (FLOSS).
Trial registration {2a and 2b}.	ClinicalTrials.gov**;** NCT06315855; 03/11/2024
Protocol version {3}	8/13/2024; Version 2
Funding {4}	George E. Richmond Foundation
Author details {5a}	1. Georgetown Lombardi Comprehensive Cancer Center, 1010 New Jersey Ave SE, Washington DC, 20,003 2. Department of Epidemiology, Graduate School of Arts and Sciences, Georgetown University, 3520 Prospect St NW, Ste 400, Washington, DC 20057
Name and contact information for the trial sponsor {5b}	Georgetown University
Role of sponsor {5c}	Funders are not involved in study design, collection, management, analysis, interpretation of data, writing of the report, or the submission of the report for publication. Funders do not have ultimate authority over any of these activities.

## Introduction

### Background and rationale {6a}

Disparate rates of poor oral health exist among cancer survivors—in part due to oral complications from cancer treatments (e.g., chemotherapy and oral mucositis). Over 40% of cancer survivors develop oral mucositis (e.g., mouth sores) during treatment. Oral mucositis and other oral complications can lead to treatment delays negatively impacting treatment efficacy and quality of life in cancer survivors [1]. Despite their bidirectional relationship [2], few studies have investigated oral and systemic health outcomes in cancer survivors from a integrative lens. We briefly review the literature on oral and systemic health within cancer survivorship, with the aim of elucidating the potential efficacy of oral health treatment in improving cancer survivorship.

#### Oral and systemic health in survivors

The oral cavity is a microcosm of the human system [hereto referred to as the oral microbiome], showing signs of several systemic health conditions [2]. The oral microbiome contains upwards of 2,000 microorganisms that symbiotically promote the oral health of an individual (e.g., homeostasis). A disruption to oral homeostasis, (i.e., oral dysbiosis) can lead to the development of oral disease (e.g., periodontal disease), by way of inflammation [3]. Inflammation is one central biological pathway by which oral health impacts systemic health, as oral microbiota may enter the circulatory system (via distressed tissue), respiratory system (through aspiration) or gut system (through the digestive tract) [3]. For cancer survivors, cancer therapies disrupt oral homeostasis leading to tissue destruction and inflammation. Periodontitis is a chronic inflammatory disease that not only increases cancer incidence risk by 122% [4] but is also prevalent in cancer survivors [5]. Vargas-Villafuerte and colleagues (2016) examined inflammation among patients with and without breast cancer following periodontal treatment. Results indicated that treatment of periodontal disease substantially improved inflammation (as evidenced by reduced CRP levels) in patients without cancer; however, periodontal treatment in cancer survivors did not yield a significant reduction in inflammation, suggesting that cancer survivors may need additional oral health promotion and treatment beyond standard of care for non-cancer patients [6].

In addition to inflammation as a biological pathway impacting systemic health in survivors, modifiable behavioral risk factors (e.g., diet) link oral and systemic health outcomes by altering the oral microbiome. For example, data from NHANES 2005–2016, show that cancer survivors report suboptimal diet quality [7], which is linked with increased risk for chronic comorbidities (e.g., diabetes) and mortality [8]. Nutrient-rich and nutrient-diverse diets that are low in sugar, high in fiber, and high in omega-6 to omega-3 fatty acid ratio can reduce risk of periodontal disease by diversifying oral microbiota and aiding in maintaining oral homeostasis [9]. Addressing biological and behavioral pathways that inform oral and systemic health in cancer survivors has potential to improve overall health and quality of life in cancer survivors.

#### Oral health and quality of life

Oral health problems can significantly impact quality of life (i.e., oral health related quality of life) yielding functional limitation, physical pain, psychological discomfort, physical disability, social disability etc. [10]. Jardim and colleagues examined oral health and oral health-related quality of life in a sample of 151 breast cancer survivors and found that xerostomia (i.e., dry mouth) and 3 or more filled teeth were associated with a 2.92-fold and 1.98-fold greater odds of having a lower oral-health related quality of life, compared to women without dry mouth and a smaller number of filled teeth [11]. These findings demonstrate that oral health promotion has potential to improve quality of life in cancer survivors (e.g., daily functioning including chewing, swallowing).

Taken together, studies demonstrate an association among oral health (e.g., treated periodontal disease) cancer incidence and morbidity, and quality of life in cancer survivors. Current literature and evidence-based trials to improve oral health outcomes in cancer survivors have predominantly focused on head and neck cancer sites [12]. There is an urgent need to address the oral health needs of non-head and neck cancer survivors in order to improve quality of life.

### Objectives {7}

To enhance quality of life among cancer survivors, solutions that address risk factors integral to oral and systemic health are needed. Although basic oral protocols are recommended as part of oncology patient care [1], cancer survivors may benefit from additional support outside of the clinical setting to promote long-term oral and systemic health. Oral telehealth—technology-supported oral health promotion—is an effective and accessible oral health promotion tool used outside of the clinic. Oral telehealth has shown efficacy in reducing plaque and gum swelling [8], yet is an underutilized tool for oral health promotion particularly among cancer survivors. Few studies have systematically examined the efficacy of oral health promotion in improving systemic health outcomes in cancer survivors [2, 7]; and no studies—to our knowledge—have used a randomized controlled trial design. Moreover, prior oral telehealth studies have not been theory-driven and lack fidelity monitoring of treatment protocol [1].

### Trial design {8}

Facilitated Lessons on Oral and Systemic Health in Survivors (FLOSS) is a 2-arm, parallel group, 1:1 allocation, superiority randomized controlled trial.

## Methods: participants, interventions and outcomes

### Study setting {9}

Study procedures including assessments will take place in community-based research office. Intervention delivery will occur via telehealth platform, Teledent. Data collection will occur in Washington, DC Metropolitan area within the Mid-Atlantic region of the US.

### Eligibility criteria {10}

The study population will comprise 100 unselected cancer survivors, recruited via established community-based approaches. “Unselected’ cancer survivors refer to individuals not selected based on cancer type. With the exception of head and neck cancer, all other cancer sites are eligible for this study. Survivors will be identified via cancer survivorship groups and established community-based approaches (e.g., community outreach programming). The inclusion criteria are: (1) male and female cancer survivors who ≥ 6 months after completion of primary therapy for cancer treatment; (2) greater than 25 years of age, and less than 75 years of age; (3) all race/ethnic groups; and (4) own a smart phone. The exclusion criteria include the following: (1) all head and neck/oral cancers; (2) less than 25 years of age and greater than 75 years of age; (3) recurrence/second cancers; and (4) unable to provide informed consent.

### Who will take informed consent? {26a}

Trained research personnel, including a postdoctoral research fellow and graduate students will obtain informed consent from potential trial participants.

### Additional consent provisions for collection and use of participant data and biological specimens {26b}

Eligible participants are informed of biospecimen collection, handling, and storage as part of the consenting procedure for all study activities. Study personnel inform participants that saliva samples will be self-collected and blood samples will be collected by a trained phlebotomist. In addition, study personnel share that samples will be de-identified, transported by a courier, and processed and stored at the Tissue Culture and Biobanking Shared Resource at Georgetown University. Written consent is obtained on the consent form for the collection of biospecimen. At the time of biospecimen collection, study personnel obtain verbal consent from participants for both saliva and blood samples. The collection of biospecimen for each participant is documented by study personnel and saved within the participant record.

## Interventions

### Explanation for the choice of comparators {6b}

Oral telehealth has shown clinically significant reductions in plaque, gingival inflammation, and the incidence of mouth sores (i.e., white lesions from oral mucositis), compared to conventional approaches [13]. Thus, its selection as a choice comparator is justified. While prior interventions have used asynchronous approaches, many have lacked personalization. This trial uses a synchronous approach to provide tailored feedback to aid individual participants in attaining the oral health goals.

Participants in the usual care arm will receive biweekly text messages which provide general health information and recommended practices about oral and systemic health.

### Intervention description {11a}

The intervention is grounded in social-cognitive theory, which posits that human behavior is mutually-influenced by person- and environmental-level factors [9]. The FLOSS project is a 6-month telehealth intervention (THI) that provides education on cognitive, behavioral and environmental-level factors that inform oral and systemic health. Participants randomized to THI arm will engage in 6, monthly 1-h virtual sessions with a facilitator training in motivational interviewing. Each session will contain: 1) a participant check-in, 2) content designed to build oral health literacy and self-efficacy, 3) and goal-setting and implementation planning. Participants will complete module handouts on-paper or electronically that reinforces module content. Additionally, participants will also receive biweekly tailored text messages aligned with monthly module content. Post-intervention follow-up will occur at 6- and 12-month post-randomization. See lineup of module topics below:

**Table:** 

Module Topic	Content
1. Connecting Oral and Systemic Health	Relationship between oral health and cancer outcomes Proper Brushing Technique Using Bass Method
2. Social Determinants of Oral Health	Common risk factors linking oral and systemic health Proper Flossing Technique
3. Diet and Oral and Systemic Health	Sugar and Oral Microbiome Healthy Hydration
4. Smoking and Alcohol and Oral and Systemic Health	Effects of Smoking on Oral and Systemic Health
5. Cancer Survivors, Oral and Systemic Health	Unique Oral Health Experiences of Cancer Survivors Managing Pain/Discomfort
6. Implementation for Long Term Maintenance	Completing Action Plan for Behavior Maintenance Celebrating Successes Exit Interview

### Criteria for discontinuing or modifying allocated interventions {11b}

Participants can withdraw from either study arm voluntarily. Participants randomized to the telehealth intervention group who wish to withdraw from the study will receive the intervention module materials via email or print. We will collect assessment data from participants who discontinue the intervention.

### Strategies to improve adherence to interventions {11c}

Fidelity monitoring is ongoing to assess treatment fidelity. Facilitators whose adherence to protocol is suboptimal will undergo additional protocol training.

### Relevant concomitant care permitted or prohibited during the trial {11d}

Treatment for cancer is prohibited during the trial. Participants cannot be undergoing treatment.

### Provisions for post-trial care {30}

This study does not have provisions for post-trial care. Participants will receive a 6-month report of progress pre- and post-intervention.

### Outcomes {12}

#### Primary outcomes

Primary outcomes of interest are related to oral health, including image-rated oral mucositis image-rated gingival inflammation and quality of life. We will also capture self-reported oral mucositis and gingival inflammation via survey (using Qualtrics Online Survey Platform [18]). Our target endpoint is 6-months, post-randomization.

##### Intraoral Imaging

All participants, prior to randomization will undergo an intraoral imaging assessment. Intraoral imaging will be conducted on all study participants at the time of their baseline in-person visit using Mouthwatch® intraoral cameras. Photos of the mouth, teeth, and gums will be captured and saved in individual patient files for research and data purposes only. Images will be captured at 6- and 12-month follow-up assessment visits. We will have a dental consultant review the images and provide summary data. The World Health Organization oral mucositis toxicity scale will be used to assess oral images. The WHO Oral Toxicity Scale rates the anatomical, and function aspects of oral mucositis. The severity of oral ulcers and ability to consume food and liquid is reported on a scale of 0 (no presentation of symptoms) to 4 (not able to tolerate food or liquid diet). Higher scores indicate worse oral mucositis symptomology. To assess gingival inflammation, the Modified Gingival Index will be applied to intraoral images. We will rate inflammation of the gums on a 5-point Likert Scale ranging from 0 (absence of inflammation) to 4(severe inflammation, marked redness, edema. Higher scores indicate more severe inflammation.

#### Self-report oral health measures

##### Oral Mucositis

The 15-item Patient Reported Oral Mucositis Scale (PROMS) will be used to examine participant experiences of oral mucositis. Respondents indicate experiences with oral mucositis on a variety of scales ranging from no experience of a mucositis-related symptoms to complete experience of symptoms. Example items include: *In the past week, have you had difficulty eating hard foods because of mouth sores.* In a validation sample, the PROMS scale was internally consistent (*α* = 0.86).

##### Oral health related quality of life

We will assess oral health-related quality of life using two measures. The Oral Health Impact Profile-14 is a 14-item scale designed to assess respondent’s perceptions of the impact of their oral conditions on their wellbeing.[19] Respondents indicate their agreement with a statement (Yes/No). Example items include: *“Do you feel your sense of taste has worsened because of problems with your teeth, mouth, or dentures?*” and” Have *you had to interrupt meals because of problems with your teeth, mouth, or dentures?”.* In a validation sample, the OHIP-14 has strong internal consistency (α = 0.88). Additionally, the 15-item European Organization for Research and Treatment of Cancer (EORTC)-Oral health module will be used. This questionnaire is designed to assess the conditions of informing oral health (e.g., bleeding gums, lip sores, taste change, sensitivity to food and drink). On items 1–11, respondents indicate agreement on a 4-point Likert Scale ranging from 1 (Not at all) to 4 (Very Much). On items 12–15, respondents indicate dichotomous agreement (Yes/No). Example items include, *“In the past week, have you had sores in the corner of your mouth?”* and *“Have you received any information about possible dental or mouth problems?”.* In an international validation study with cancer survivors, internal validity was acceptable (*α* = 0.79).

#### Secondary outcomes

Secondary outcomes of interest are related to systemic health and include systemic inflammation, overall quality of life, and comorbidities. Measures include collection of biospecimen, anthropometrics, and self-report measures. The target endpoint for secondary measures is 6-months, post-randomization.

#### Biospecimens

##### Saliva/DNA sample collection

Saliva samples will be collected to analyze the oral microbiome. Participants will first be instructed not to consume food or drink 30 min prior to assessment visit. Participant will self-collect (at baseline, 6- and 12-post randomization) the saliva sample (i.e., expectorate) using the Omnigene® Saliva DNA and RNA device (OMR-610). Saliva samples will be stored for up to 21 days at room temperature, and then will be stored at −20 °C. Samples will be processed by the Georgetown University Medical Center-Lombardi Comprehensive Cancer Center Tissue Culture and Biobanking Shared Resource (TCSBR) per manufacturers protocol using RNeasy Power Microbiome Isolation Kit to isolate human and bacterial DNA, and MagMax Viral/Pathogen Nucleic Acids Isolation kit to extract viral nucleic acid.

##### Blood Collection

Non-fasted blood samples will be collected to assess inflammatory markers including C-reactive protein, IL-1, IL-6, and TNF-alpha will be measured. Samples will be processed to separate plasma and buffy coat aliquots by GUMC-Lombardi TCBSR within two hours and stored at − 80 °C.

#### Anthropometrics

Height: Participant height will be captured by a trained assessor, using a calibrated stadiometer. Height will be measured in centimeters and rounded to nearest tenth. Weight: Participant weight will be captured by a trained assessor, using a calibrated scale. Weight will be measured in kilograms and rounded to nearest tenth. Waist Circumference: Using tape measure, a trained assessor will measure the smallest portion of the participant’s waist in centimeters, rounding to the nearest tenth. Hip Circumference: Similar to waist circumference, hip circumference will be captured by a trained assessor measuring the widest part of the participant’s waist, rounding to the nearest tenth.

#### Self-report systemic health measures

##### Health-related quality of life

The EORTC Quality of Life-C30 [20] will assess health-related quality of life. Respondents will report on functioning (including physical, cognitive and emotional), and presenting symptoms (e.g., fatigue, pain, nausea and vomiting, sleep disturbance, appetite, etc.). On items 1–28, respondents will indicate agreement on a 4-point Likert scale ranging from 1(Not at all) to 4(very Much). Items 29 and 30 utilize a 7-point Likert scale ranging from 1(very poor) to 7(excellent). Example items include *“Do you have trouble taking a long walk?”* and “*During the past week, were you limited in doing either you work or other daily activities?”.*

##### Health behaviors

Dietary intake (including sugar-sweetened beverages [SSBs]), alcohol use, and smoking behaviors will be assessed using items from the widely-utilized Behavioral Risk Factor Surveillance Survey [21]. On dietary intake items, respondents will indicate their consumption of vegetables, fruits and fiber/whole grains. Item ratings include less than once/week; once per week, 2–3 times per week, 4–6 times per week, once/day, and more than once per day. Respondents will also indicate their daily meal patterns (i.e., average number consumed, and meal skipping). An example item includes: “*How often did you eat a green leafy or lettuce salad, with or without other vegetables?”.* Sugar-sweetened beverage consumption will be captured via 3 items, which assess consumption of SSB in past 7 days, average daily consumption, and knowledge of health implications of frequent consumption of SSBs. On alcohol use items, respondents will indicate number of alcoholic drinks consumed across lifespan, and within past 30 days. Item ratings include: 0 days, 1 or 2 days, 3–5 days, 3–9 days, 10–19 days, 20–29 days, and all 30 days. Lastly, smoking behaviors will be assessed via 4 items, which assess smoking behaviors across lifespan, daily smoking behaviors, and daily number of cigarettes consumed. Item ratings are similar to those of alcohol use.

##### Clinical characteristics

Participants will report cancer treatment history via 11 items, which assess cancer type, treatment, and overall cancer care. Participants will also indicate the presence of prior or existing health conditions (oral and systemic) including but not limited to: diabetes, stroke, lung disease, stomach ulcers, bleeding gums, sensitive teeth, etc.

##### Demographics

Participants report on age, gender, racial-ethnic identification, marital status, education, employment, income, zip code, preferred contact information, access to health care, as well as dental care history (Table 1).

**Table 1:** Timepoints of measurements

**CONSTRUCT**	Timepoint (Months)		
	0	6	12
*Anthropometrics*			
Height	X	X	X
Weight	X	X	X
Waist Circumference	X	X	X
Hip Circumference	X	X	X
*Biospecimen*			
Saliva Sample	X	X	X
Blood Sample	X	X	X
*Imaging*			
Intraoral Image	X	X	X
*Self-Reported Oral Health*			
OHIP-14	X	X	X
Oral Health-Related Quality of Life (OH-15)	X	X	X
Quality of Life (OH-15)	X	X	X
*Behavioral*			
Smoking and Tobacco Use	X	X	X
Alcohol Use	X	X	X
Diet	X	X	X
Physical activity	X	X	X
Oral-health related self-efficacy	X	X	X
Dental related anxiety	X	X	X
*Other*			
Demographics	X	X	X
Access to HealthCare	X	X	X
Cancer Treatment	X	X	X

Analysis Metric: Change from baseline

### Participant timeline {13}(Table 2)

**Table 2:**
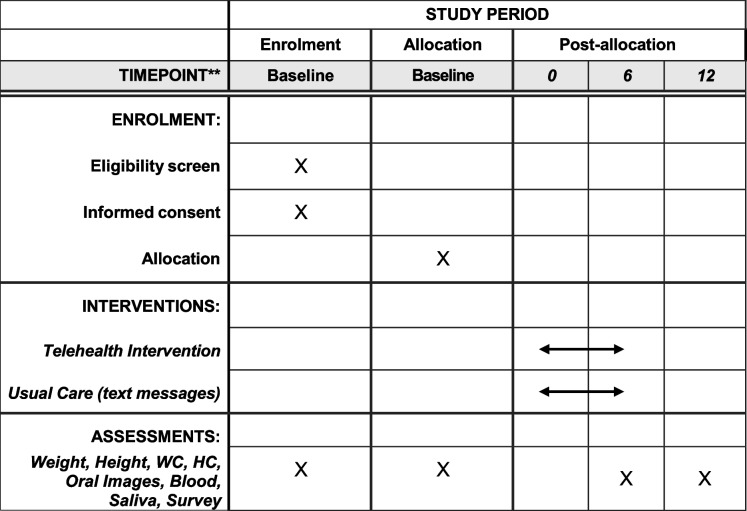
Schedule of enrollment, intervention and assessments for FLOSS Project

### Sample size {14}

A power analysis was conducted using effect sizes for telehealth-delivered, psycho-behavioral interventions in cancer survivors. A 6-month study by Abrahams et al. (2017) examining the efficacy of an internet-based cognitive behavior therapy intervention for breast cancer survivors reported mean quality of life of 77.1 (SD = 16.5) and 63.9 (SD = 20.1) for intervention arm and care as usual arm, respectively. This translates to an effect size (standardized mean difference) of 0.71 standard deviations. Based on this finding, we determine sample size for selected power levels and the standardized mean difference (Cohen’s *d*) between the telehealth and usual care arms of the study in the neighborhood of 0.71 standard deviations. Table 3 below presents sample sizes per arm required for selected values of Cohen’s *d* at level of significance, α = 0.05.

**Table 3:** Sample sizes per group for selected power and effect size

Power	Effect size (Standardized mean difference)					
	0.30	0.40	0.50	0.60	0.70	0.80
80%	175	99	64	45	33	26
90%	234	132	85	59	44	34

For this study, we will recruit 100 subjects (50 subjects per arm) and expect to detect a medium effect size of approximately 0.60 standard deviations (Cohen’s *d* = 0.60) with 80% power, allowing for an attrition rate of 10%. Of note, sample size will not be adjusted during this trial. Results from this trial will help determine sample size for a larger trial.

### Recruitment {15}

Participants will be actively recruited from community settings. Study personnel will recruit through the organizational networks of community partners (e.g., survivorship and cancer support organizations, newsletters, flyers advertisements) and targeted outreach in the Washington Metropolitan area. Community partners will aid recruitment by directing eligible participants (as indicated on study promotional materials) to online screener form or study coordinator via telephone. Prospective participants will also be identified through cancer survivorship groups and research registries. Individuals who preliminarily meet eligibility criteria will be contacted via phone or email by study personnel.

## Assignment of interventions: allocation

### Sequence generation {16a}

Following completion of baseline assessments, participants will be randomly assigned, in a 1:1 ratio, to either the THI or UC arm. A computer-based randomization sequence will be generated by the first author. Intervention allocations will be placed in a password protected folder and stored electronically on university-approved servers (i.e., Box). The study coordinator will examine the randomization allocation on the day of the consent visit and inform participants of their group allocation at the conclusion of their baseline visits and assign participants to group based on these numbers.

### Concealment mechanism {16b}

Intervention allocations (identified by study ID numbers) will be entered into a document and placed in a password protected folder and stored electronically on university-approved servers (i.e., Box).

### Implementation {16c}

The study coordinator and trained research assistants will enroll participants. The study coordinator will verbally inform participants of their intervention allocation following baseline assessments.

## Assignment of interventions: blinding

### Who will be blinded {17a}

The study PI and data analysts will be blinded after assignment to interventions.

### Procedure for unblinding if needed {17b}

The Study PI and data analysts will remain blinded to the randomization assignments of participants.

## Data collection and management

### Plans for assessment and collection of outcomes {18a}

Assessors will undergo research ethics training, subject-matter training (i.e., link between oral and systemic health), trial-specific training (facilitated by the study coordinator)—including mock assessments.

### Plans to promote participant retention and complete follow-up {18b}

Participants receive compensation at each assessment timepoint totaling, $100. Participants will also receive a progress report with detailed feedback from a dental consultant on their oral health (including, baseline and 6-month timepoints).

### Data management {19}

Anthropometric data collected from participants will be entered directly into participant file (i.e., paper file). Following this, data will be entered by trained research assistant directly into an electronic datafile stored on secured servers. Oral images will be renamed (per protocol) and downloaded to university-approved servers. Data will be checked by study personnel on a monthly basis to ensure all data is accurately entered and complete.

### Confidentiality {27}

All electronic data will be stored on university-approved secure servers (GU Box). Subjects will be identified by a unique ID number. Links between ID numbers and identifying information, such as telephone numbers, will be password protected. Only study personnel will have access to study data. Data will be directly stored in a database with daily network back-ups.

Blood and saliva samples will be identified with a Unique ID and only evaluated for research purposes. Data linking keys will be stored in a separate database.

All data collection reports and summaries will be kept in a locked file in the Study Coordinator’s office at the research site at Georgetown University’s Lombardi Comprehensive Cancer Center. Data collection instruments will be administered through a HIPPA Compliant online survey platform, Qualtrics.

Standard safeguards to protect the data will be followed. All data will be kept in locked file drawers. Access to data files that contain identifying information will be secured with a password filing system and will be restricted to only approved project staff. All project file cabinets and databases will be secured and locked when not in use.

Audio-recordings of telehealth sessions will be identified by Unique ID, time and date of the session. Audio files will be stored securely on university-approved secure servers (e.g., GU Box).

### Plans for collection, laboratory evaluation and storage of biological specimens for genetic or molecular analysis in this trial/future use {33}

Biospecimens will be transported via private courier and stored at the Lombardi Cancer Center at the Tissue Culture and Biospecimen Resource Shared Resource for processing. Data and specimens will be stored for 10 years after the completion of the primary analyses.

## Statistical methods

### Statistical methods for primary and secondary outcomes {20a}

Demographic and other baseline data including cancer-related clinical characteristics will be listed and summarized descriptively by intervention arm (THI and UC). Categorical data will be presented as frequencies and percentages. For continuous data, mean, standard deviation, median, minimum, and maximum will be presented. For selected parameters, 25th and 75th percentiles will also be presented.

Intention to treat (ITT) analysis will be performed for all participants who enrolled and randomly allocated to one of the clinical trials arms. Descriptive statistics will be used to summarize the original scores for the outcomes (oral and systemic health outcomes), as well as change from baseline, at each scheduled assessment timepoint for the THI and UC group. Additionally, change from baseline in the scale and subscale values at the time of each assessment will be summarized. Participants with an evaluable baseline score and at least one evaluable post baseline score during the treatment period will be included in the change from baseline analyses. The significance of the difference in mean scores between the two arms will be assessed using paired t-test or Wilcoxon signed-rank test. In addition, for each outcome measure, analysis of covariance (ANCOVA) models will be fit to evaluate the difference in mean scores at each timepoint post-baseline with baseline scores as concomitant variables. Repeated measures ANOVA models will be used to examine mean differences in the scores of image-rated oral mucositis, image rated gum inflammation, blood biomarkers of systemic inflammation, and oral and overall quality of life between the THI and UC arms. The differences in least square means between the treatment arms and corresponding 95% confidence interval at selected timepoints will be presented. The Bonferroni method will be used to adjust models for multiple testing.

### Interim analyses {21b}

No interim analysis will be conducted.

### Methods for additional analyses (e.g. subgroup analyses) {20b}

We will not conduct subgroup analyses.

### Methods in analysis to handle protocol non-adherence and any statistical methods to handle missing data {20c}

We will employ intent-to-treat method for data analysis to address missing data and protocol nonadherence. In addition, we will examine patterns of missing data over the intervention period, and use multiple imputation to address incomplete data.

If participants receive a new or recurrent cancer diagnoses during the intervention, they will be asked to report it to study personnel. The participant will be able to continue study procedures (i.e., intervention and assessments), but their data will not be included in the final analyses.

### Plans to give access to the full protocol, participant level-data and statistical code {31c}

Per our informed consent form, participant-level data will not be available for analysis. Statistical code will be made available upon request.

## Oversight and monitoring

### Composition of the coordinating centre and trial steering committee {5d}

The Trial Steering Committee consists of a postdoctoral fellow who acts as a study coordinator and co-investigator; 3–4 graduate-level research assistants, and 2 clinical personnel (phlebotomist, exercise physiologist).

### Composition of the data monitoring committee, its role and reporting structure {21a}

This study presents minimal risk and thus no data monitoring committee is needed.

### Adverse event reporting and harms {22}

Although unanticipated given the primarily educational nature of this intervention, any adverse events would be reported to the IRB within 24 h.

### Frequency and plans for auditing trial conduct {23}

Fidelity monitoring is ongoing to assess treatment fidelity. Facilitators whose adherence to protocol is suboptimal will undergo additional protocol training. The study coordinator will meet with the dental consultant a minimum of every 3 months to receive feedback on the quality of intraoral images.

### Plans for communicating important protocol amendments to relevant parties (e.g. trial participants, ethical committees) {25}

Changes to protocol will be reported to the IRB within the week of identification of a need for an amendment. Changes that need to be communicated to trial participants will be sent via email, with a brief note sent via text.

### Dissemination plans {31a}

Research personnel will disseminate findings via publication and presentations at national conferences, and local seminars/workshops. A detailed report will be prepared for the study funder/sponsor. A brief infographic will be prepared to share with participants via email. Study findings will also be shared in the Office of Minority Health and Health Disparities Research Community Newsletter.

## Discussion

THI Participants will receive instructions on use of the telehealth platform, and will be offered technical assistance if needed.

## Trial status

Protocol version 2.0- 08/13/2024. Recruitment start date: May 2024; Recruitment end date: June 2026.

## Data Availability

Only study personnel will have access to data.

## Abbreviations

THI: Telehealth Intervention
UC: Usual care
ITT: Intent to treat analysis
